# A time frame permissive for Protein Kinase D2 activity to direct angiogenesis in mouse embryonic stem cells

**DOI:** 10.1038/srep11742

**Published:** 2015-07-07

**Authors:** Martin Müller, Jana Schröer, Ninel Azoitei, Tim Eiseler, Wendy Bergmann, Ralf Köhntop, Qiong Lin, Ivan G Costa, Martin Zenke, Felicitas Genze, Clair Weidgang, Thomas Seufferlein, Stefan Liebau, Alexander Kleger

**Affiliations:** 1 Department of Internal Medicine I, Ulm University, Ulm, Germany; 2 Department of Cell Biology, Institute for Biomedical Engineering, RWTH Aachen University Medical School, Aachen, Germany; 3 Department of Urology, Ulm University, Ulm, Germany; 4 IZKF Computational Biology Research Group, RWTH Aachen University Medical School, Aachen, Germany; 5 Institute of Neuroanatomy, Eberhard Karls University Tuebingen, Tuebingen, Germany

## Abstract

The protein kinase D isoenzymes PKD1/2/3 are prominent downstream targets of PKCs (Protein Kinase Cs) and phospholipase D in various biological systems. Recently, we identified PKD isoforms as novel mediators of tumour cell-endothelial cell communication, tumour cell motility and metastasis. Although PKD isoforms have been implicated in physiological/tumour angiogenesis, a role of PKDs during embryonic development, vasculogenesis and angiogenesis still remains elusive. We investigated the role of PKDs in germ layer segregation and subsequent vasculogenesis and angiogenesis using mouse embryonic stem cells (ESCs). We show that mouse ESCs predominantly express PKD2 followed by PKD3 while PKD1 displays negligible levels. Furthermore, we demonstrate that PKD2 is specifically phosphorylated/activated at the time of germ layer segregation. Time-restricted PKD2-activation limits mesendoderm formation and subsequent cardiovasculogenesis during early differentiation while leading to branching angiogenesis during late differentiation. In line, PKD2 loss-of-function analyses showed induction of mesendodermal differentiation in expense of the neuroectodermal germ layer. Our *in vivo* findings demonstrate that embryoid bodies transplanted on chicken chorioallantoic membrane induced an angiogenic response indicating that timed overexpression of PKD2 from day 4 onwards leads to augmented angiogenesis in differentiating ESCs. Taken together, our results describe novel and time-dependent facets of PKD2 during early cell fate determination.

The protein kinase D (PKD) family belongs to the calcium-/calmodulin-dependent protein kinase superfamily^1^ and comprises the three evolutionary conserved isoforms, PKD1, −2 and −3^2^. PKDs are serine threonine kinases which can be activated by various stimuli, including phorbol esters, G-protein-coupled receptors and reactive oxygen species (ROS)^2^,^3^. PKDs act as prominent downstream targets of PKCs, especially the novel PKCη^4^,^5^. The PKD family plays a role in DNA synthesis, proliferation, cell survival, adhesion, invasion/migration and motility. Furthermore, PKDs regulate protein transport by facilitating the fission of budding vesicles from the trans-Golgi network^6^,^7^,^8^,^9^,^10^. Despite their broad expression in the early embryo, the role of PKD isoforms during development and cell fate choice is largely elusive^11^,^12^. Herein, PKD2 has been recently shown to regulate cardiac valve formation^13^ but also erythropoiesis^14^. However, only a handful of studies report on the expression of PKDs in various stem cell populations. We recently demonstrated that distinct PKD isoforms, dominated by PKD2, are expressed in undifferentiated myoblasts and regulate their differentiation^15^. Similarly, a BMP-PKD2 axis regulates osteoblast differentiation from human mesenchymal stem cells^16^. However, PKD2 activity is not only present in normal stem cells but also in tumour stem cells as shown for CD133(+) glioblastoma-initiating cells^17^. A recent study identified PKD1 as an anti-differentiate, pro-proliferate signal in the skin tissue^18^. This observation is not only limited to physiological skin formation but also to cancer initiation. The expression of CD34 in cutaneous cancer stem cells is required for stem cell activation and tumour formation. Furthermore, PKD1 was found to be strongly expressed in CD34(+) cells and that inhibition of PKD1 could be preventive in skin cancer development^18^.

As one of the early events during gastrulation, definitive endoderm (DE) and anterior mesoderm derivatives, including cardiovascular and head mesenchyme progenitors, are generated from a transient precursor cell population located in the region of the anterior primitive streak. This cell population is commonly referred to as mesendoderm giving rise to mesoderm and endoderm and is marked by the expression of marker genes such as Brachyury (T) and Foxa2^19^,^20^,^21^. Soon afterwards, the development of the circulatory system is initiated from a common multipotent progenitor cell type, the so-called hemangioblast. This process of *de novo* formation of blood vessels is called vasculogenesis and is prevalent in the mouse embryo until E8.5. Vasculogenesis is accompanied by a complementary process called angiogenesis, an event that defines vessel formation from pre-existing endothelial cells that undergo sprouting and that is shown to commence in the embryo at E9.5^22^. Various laboratories, including ours, have delineated the role of PKDs during physiological and tumour angiogenesis^23^,^24^,^25^,^26^,^27^,^28^. In fact, recent data indicate that in endothelial cells PKD2 is the predominant PKD isoform that is required for proliferation, migration, *in vitro* angiogenesis and expression of vascular endothelial factor receptor-2 (VEGFR2) as well as fibroblast growth factor receptor-1 (FGFR1)^26^. Moreover, our group identified PKD2 as a novel, essential mediator of tumour cell-endothelial cell communication^29^ and as a critical modulator of hypoxia-induced VEGF expression/secretion by the tumour cells^30^. Other recent studies from our laboratory described PKD1 and −2 isoform-selective effects on cancer cell invasion and angiogenesis^17^,^31^,^32^.

The only data linking PKDs to vasculogenesis come from a recent study in zebrafish. Herein, PKD1 deletion moderately reduced the formation of the intersomitic vessels and the dorsal longitudinal anastomotic vessel. In addition, the formation of the parachordal lymphangioblasts, a precursor for the developing thoracic duct, is perturbed upon PKD depletion. By contrast, PKD induced tumour angiogenesis in zebrafish xenografts^33^. This indicates a time-restricted PKD-responsive window during distinct developmental stages and a strong PKD effect during angiogenesis. However, such a hypothesis has never been explored due to the lack of appropriate model systems.

Pluripotent stem cells represent a powerful tool to investigate embryonic development in mouse and human^34^,^35^,^36^,^37^. Moreover, these cells provide a unique platform for dissecting the distinct mechanisms underlying pluripotency and subsequent lineage commitment^37^. Given the high corroboration between embryonic development (*in vivo*) and pluripotent stem cell differentiation (*in vitro*), various factors can be investigated in a time- and stage-dependent manner regarding their impact on cell fate determination, differentiation and/or tissue formation^21^,^38^,^39^,^43^.

In this study, we examined the contribution of PKD2 during cell fate determination and cardiovascular lineage formation. To investigate the timed PKD2 activation during ESC differentiation, we used mouse embryonic stem cells (ESCs) and induced pluripotent stem cells (iPSCs) for *in vitro* cell culture experiments as well as for *in vivo* assays with a CAM (chorioallantoic membrane) xenograft. PKD2 is dynamically expressed/activated during the first days of differentiation. Functionally PKD2 represses mesendoderm formation and subsequent cardiovascular lineage commitment when activated during germ layer segregation. At later stages, PKD2 promotes exclusively the vascular lineage. In line with these findings, a genetic loss-of-function approach based on the expression of a kinase-dead variant showed a reciprocal differentiation pattern as evident by an increase in mesendodermal differentiation. Thus, our data define for the first time a PKD2-responsive time-window to drive predominantly angiogenesis instead of vasculogenesis.

## Experimental Procedures

### Ethics statement

All animal experiments were performed in compliance with the guidelines for the welfare of experimental animals issued by the Federal Government of Germany, the National Institutes of Health and the Max Planck Society. The experiments in this study were approved by the review board of the Land Baden-Wuerttemberg and the ethics committee of Ulm University No.:o.197.TschB:K/W/O/H.

### Generation of iPKD2-ESCs

One day before the nucleofection procedure, A2lox.cre cells were incubated with 1 mg/ml of doxycycline to induce the recombination by Cre-recombinase. ESCs were transfected using the Nucleofector Technology (Lonza, USA) according to the manufacturer’s procedures. 10 μg DNA (iPKD2-p2lox vector) was used to transfect 5 million parental A2lox.cre ESCs. After transfection cells were plated on neomycin-resistant, Mitomycin-C (Sigma, USA) inactivated MEFs. Two days later cells were subjected to selection with neomycin (400 μg/ml) for additional 7 to 10 days. Several clones were picked, expanded and analysed for correct recombination of the HPRT locus using the “lox in” PCR.

### Generation of induced pluripotent stem cells (iPSCs) from PKD2-kinase-dead-mice

Mouse embryonic fibroblasts (MEFs) were isolated according to standard protocols from wild type C57B6 mice (WT) and from a previously reported mouse strain in C57B6 background, where the two critical serine residues (Ser 706, 710) of PKD2 (PKD2-KD) have been replaced by alanine leading to the expression of a kinase-dead mutant. This mouse strain lacks the catalytic activity of PKD2 and recapitulates the phenotype of a PKD2 gene trap line^40^,^41^. Reprogramming virus was generated according to standard protocols as described previously^34^,^37^,^42^. 100,000 WT and PKD2-KD MEFs per well of a 6well-plate or 40,000 cells per well of a 12well-plate, respectively, were seeded one day before infection with virus. Next day, 8 μl per well of a 6well-plate or 4 μl per well of a 12well-plate, of 100 × concentrated virus (=3,5 × 10^7^ proviral hOSKM copies/μl) were added to the cells as a master mix. After 8 h of incubation at 37 °C, medium was changed to ES-Feeder and refreshed every day. After one week, infection rate was assessed by FACS analysis (data not shown). At day 7, medium was changed to ESC culture conditions and changed every day. ESC culture conditions have been described previously^43^, but for reprogramming instead of fetal calf serum, knock out serum replacement (Invitrogen, Germany) was used. On day 13, cultures were fixed and stained for alkaline phosphatase (AP)-expression according to standard protocols. Several independent iPSC clones derived from WT and PKD2-KD MEFs were randomly picked based on typical ESC like morphology at day 13 of reprogramming. After expansion of individual clones, pluripotency analysis was carried out at early passage (1–5). All analysed clones were stained positive for pluripotency markers and capable to *in vitro* differentiate to the three germ layers.

### Cell Culture

Mouse embryonic fibroblast (MEF) feeder cells were cultured in DMEM supplemented with 10%, FCS (PAA, Austria), 1% Penicillin/Streptomycin, 2 mM GlutaMax (Invitrogen, Germany), 1% Non-Essential Amino Acids (NEAA; Life-technologies, USA), 1 mM Sodium Pyruvate (Invitrogen, Germany), 1% β-Mercaptoethanol (Millipore, Germany) and 0,05 mg/ml Vitamin C (Sigma, Germany) in humidified atmosphere containing 5% CO_2_ at 37 °C^21^.

mESCs and miPSCs were cultured in Knockout-DMEM (KODMEM; Life-technologies), 15% FCS (Sigma, ESC-qualified), 1% Penicillin/Streptomycin, 1% GlutaMax, 1% NEAA, 1% Sodium Pyruvate, 1% β-Mercaptoethanol and 240 U/ml leukaemia inhibitory factor (LIF; Sigma, USA). Additionally, PD0325901 (1 μM) and GSK3 inhibitor CHIR99021 (3 μM) (Selleckchem, USA) were added to the culture medium.

EB formation: Iscove’s modified Dulbecco’s medium (IMDM; Invitrogen) supplemented with 10% FCS (Lonza, USA), 1% Penicillin/Streptomycin, 1% GlutaMax, 1% NEAA and freshly prepared 450 μM Monothioglycerol (Sigma) were used for differentiation. Briefly, 600 (or as indicated in figure legends) cells per 20 μl differentiation medium were dropped on petri dish covers, placed upside down on petri dishes filled with 10 ml Dulbecco’s Phosphate Buffered Saline (DPBS; Invitrogen) and were cultivated for 2 days in hanging drops. Subsequently, embryoid bodies (EBs) were transferred onto non-adherent petri dishes and were cultivated for additional two days. On day 4, EBs were plated on 0.1% gelatine-coated 6-well dishes or cover slips for RNA or immunofluorescence analysis respectively and assayed at specific time points as described in figure legends. Doxycycline was added as indicated in figure legends.

### Genomic PCR

Genomic ESC DNA was isolated using Blood & Tissue Kit (QIAGEN, Germany). For PCR reaction, recombinant Taq DNA polymerase (Promega) was used. The reaction was performed in 25 μl containing 1 μl DNA, 0.25 μl Go-Taq 2.5 μl PCR buffer (10X), 2 μl 1.5 mM MgCl_2_, 0.5 μl of 10 mM dNTP, 0.3 μl of primer. PCR conditions for ESC analysis started with denaturation at 94 °C for 3 min followed by a primer-dependent number of cycles with denaturation at 94 °C for 30 s, annealing temperature at 61 °C for 45 s, and product elongation at 72 °C for 1 min. The following gene locus specific primer sequences have been used. To verify the recombination of the HPRT locus a so-called “lox-in” PCR was performed: LOXIN R 5′-ATA CTT TCT CGG CAG GAG CA-3′, and LOXIN F 5′-CTA GAT CTC GAA GGA TCT GGA G-3′. Genotyping of the PKD2-kinase-dead mice and iPSC lines was carried out by PCR of genomic DNA using primers 671–5′armF (5′-AGTGGCACGTTCCCCTTCAATG-3′) and 671–3′armR (5′-CTTTGCCCAATCCCTTACAGCCT-3′), producing products of 236 bp [PKD2WT (wild-type PKD2)] and 344 bp (PKD2-kinase-dead).

### Immunocytochemistry

Cells at different time points of differentiation were fixed using 4% paraformaldehyde (PFA). Samples were then subjected to treatment with NH_4_Cl and blocking with 0.3% TritonX-containing BSA before incubation with the primary antibodies. Mouse anti SSEA-1 (Santa Cruz Biotechnology, USA), 1:200, over-night at 4 °C; rabbit anti-PKD1 (Santa Cruz, USA), 1:200, 2 h at room temperature (RT); rabbit anti-PKD2 (Orbigen, USA), 1:1000, 1 h at RT; mouse anti-Oct3/4 (Santa Cruz, USA), 1:200, overnight at 4 °C; rat anti-CD31, (Becton Dickinson, USA), 1:100, 1 h RT; mouse anti α-Actin (Sigma Aldrich, Germany) 1:100, 1 h 37 °C. Samples were further incubated with fluorescence labelled secondary antibodies Alexa Fluor® 488 (green), Alexa Fluor® 568 (red), Alexa Fluor® 647 (magenta) (Life-technologies, all diluted 1:500). For germ layer-specific staining we used the chicken anti β–tubulin-III antibody ((TUBB3), Millipore, Billerica, MA), 1:1000, goat anti-human Brachyury (R&D Systems, Minneapolis, MN, USA, www.rndsystems.com), 1:100, o.N. 4°, AF2085 and goat anti-human SOX17 (R&D Systems), 1:500, o.N. 4°, AF1924. Nuclei were stained with DAPI (blue) (1:20,000). Images were captured using an upright fluorescence Zeiss Axioimager Z1 microscope and analysed using Axiovision software (Zeiss, Germany).

### Western Blot Analysis

Western Blotting was performed according to standard procedures. Whole-cell extracts (50–100 μg) prepared using IP-lysis buffer containing 10 mM Tris HCl, 5 mM EDTA, 50 mM NaCl, 50 mM NaF, and 1% Triton-X100 supplemented with Complete Protease Inhibitor Cocktail Tablets (Roche) were subjected to sodium dodecyl sulphate polyacrylamide gel electrophoresis (SDS-PAGE). Separated proteins were transferred to PVDF membranes (Millipore Corp., USA). Membranes were blocked using 5% dry milk in PBS containing 0.2% Tween 20. For subsequent washes, 0.2% Tween 20 in PBS was used. Membranes were incubated with primary antibodies over-night at 4 °C under shaking conditions. The following primary antibodies were used: anti-PKD2 (Bethyl, #A300-073 A), anti-PKD3 (Bethyl, #A300-319A), anti-PKD1 (Santa Cruz, #sc-935), anti-phospho PKD (Ser744/748) (Cell Signalling, #2054), anti-PKCδ (Santa Cruz, #sc-213) and anti-phospho PKCδ (Thr 505) (Cell Signalling, #9374). This was followed by incubation with secondary horseradish peroxidase (HRP)–coupled antibodies diluted 1:3000, 1 h at RT. Detection was performed with either ECL or ECL+ kits (Thermo scientific, USA).

### Quantitative real-time RT-PCR

qPCR analysis was performed either as one-step or two-step real-time PCR depending on the amount of analysed genes. One-step real-time qPCR was carried out with the Rotor Gene RT-PCR Cycler (Qiagen) using the QuantiTect SYBR Green RT-PCR kit (Qiagen). Each RNA preparation was tested for genomic DNA contamination by replacing reverse transcriptase with water. Internal standards (house-keeping gene) and samples were simultaneously amplified. Details have been described elsewhere^35^,^43^,^44^. For two-step real-time PCR iScript™ cDNA Synthesis Kit (Biorad, #170-8891) and SensiMix™ SYBR® No-ROX Kit (Bioline, QT650-05) have been used according to supplier’s instruction manual using 100 ng cDNA as a template. PCR conditions included denaturation at 95 °C for 20 s, followed by 50 cycles of 94 °C for 1 min and 60 °C for 20 s, then continuation with dissociation stage. QuantiTect Primer Assays were used throughout this study (Qiagen, Germany, http://www.qiagen.com/de/products/catalog/assay-technologies/real-time-pcr-and-rt-pcr-reagents/quantitect-primer-assays/).

### CAM-Assay

Shells of fertilised chicken eggs were opened on day 6 and silicon rings (5 mm in diameter) were applied onto the chorioallantoic membrane (CAM). EBs from iPKD2-ESCs were transplanted at day 4 onto CAM to generate tumours. Doxycycline (1 μM) in EB-medium has been applied ectopically on EBs at the time of transplantation and after 48 h. After 72 h EB-derived structures were harvested and photographed.

### Immunohistochemistry of CAM and EB-derived structures and quantification of vessel density

Formalin fixed tumours were embedded in paraffin using standard histological procedures. The sections were processed and stained with antibodies directed against von Willebrand factor (vWF) (1:100, DAKO). The slides were placed on the optical photomicroscope (Zeiss Axio Scope A1, Carl Zeiss Jena) and photographed with a 20x magnification objective. The visual fields in the pictures represent a side length of 450 × 340 μm on the original slide. Each picture was opened using Adobe Photoshop CC (Version 14.2.1. x64), thereafter an 11 × 8 grid was placed over the image. To quantify the amount of vessels we have counted the number of vWF positive vascular structures within the grid squares in a blinded manner. Only squares with confluent cell layering were included for statistical analysis. A total of 36 microscope sectors of 20x magnification were counted. The CAM Assay was performed in two biologically independent experiments; each experimental set up consisted of at least 5 tumours per treatment condition (at least 10 CAMs per experiment, 5 times Dox + and 5 times Dox -).

### Statistical Analysis

If not stated otherwise, SEMs are indicated by error bars. Generally levels of significance were calculated with the two-sides Student’s t test or Anova (*p < 0.05; **p < 0.01; ***p < 0.001) using Prism5 (GraphPad, USA). If not otherwise specified three biologically independent experiments done in replicates were evaluated.

## Results

### PKD2 expression in undifferentiated and differentiating embryonic stem cells

**Figure 1:**
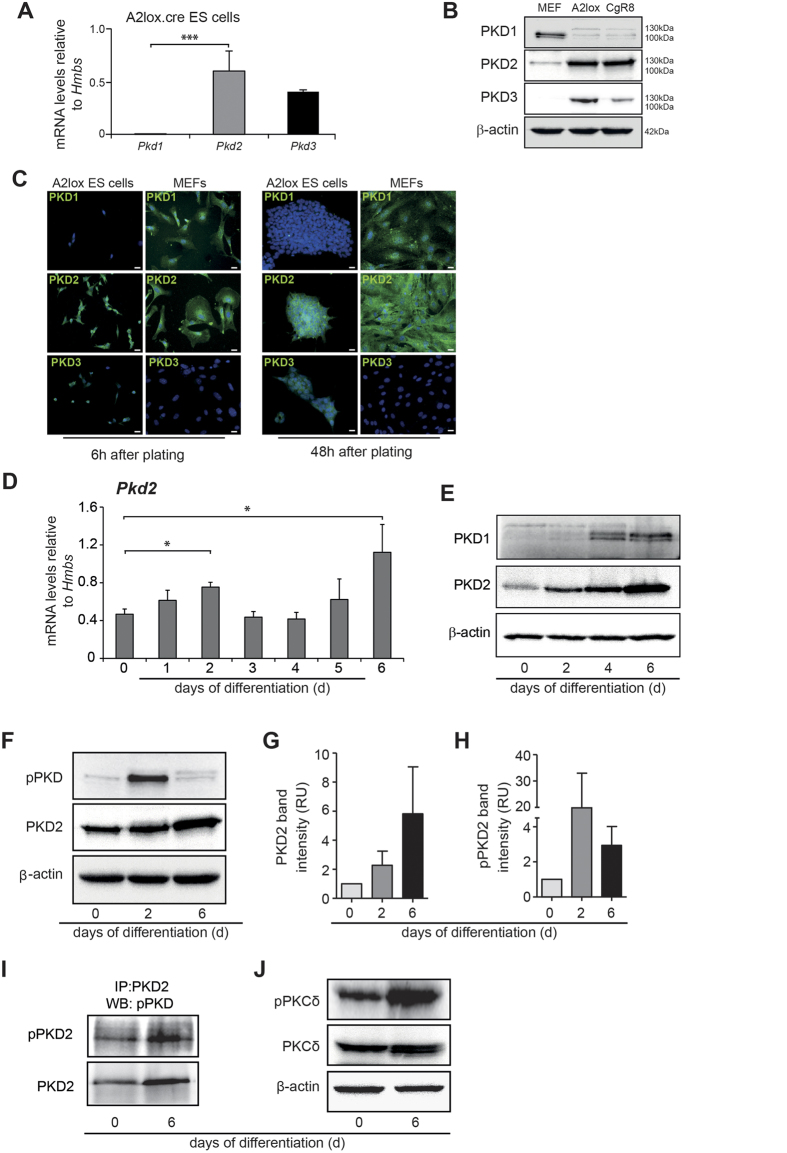
Expression of PKD isoforms in mouse embryonic stem cells. (**A**) mRNA levels of PKD 1/2/3 in undifferentiated A2lox mouse ESCs. (**B**) Western Blot analysis of PKD1/2/3 in A2lox.cre and CgR8 ESCs. MEFs served as positive control. (**C**) Immunofluorescence staining for PKD1, PKD2 and PKD3 (green) in undifferentiated A2lox mES cells 6 h and 48 h after plating under pluripotency conditions. Nuclei are stained with DAPI (blue). MEFs served as positive control. (**D**) PKD2 mRNA expression levels during EB differentiation until day 6 of ESCs. (**E**) Western blot analysis of PKD1 and PKD2 protein levels during differentiation until day6 in differentiating ES cells. (**F**) Western Blot of phospho-PKD and PKD2 indicating catalytic activity by phosphorylation within the activation loop at two conserved serine residues. (**G**,**H**) Relative band intensity of PKD2 (**G**) and pPKD2 (**H**) days 0, 2 and 6. Two experiments of three included into the quantification. (**I**) Immunoprecipitation of phospho-PKD2 (pPKD2) and PKD2 on Days 0 and 6. (**J**) Western Blot of PKCδ and phospho-PKCδ on Days 0 and 6. qPCRs were performed n = 3 in replicates. Western Blots are representative for three independent experiments. Scale bars 20 μm.

PKDs are significantly expressed in the developing mouse embryo and in particular, PKD2 shows a differentially regulated expression pattern^12^. In embryonic stem cells, PKCs (the upstream activators of PKDs) drive primitive endoderm formation, while their inhibition facilitates pluripotency^45^. However, the contribution of PKDs during cell fate determination is not clearly understood. Employing murine ESCs as a tool, we sought to characterize the role of PKDs in early stages of differentiation. First, we investigated the mRNA and protein expression of PKD1, PKD2 and PKD3 in undifferentiated mouse ESCs. As shown in Fig. 1A and B, *Pkd2* was highly expressed in ESCs, followed by *Pkd3*. *Pkd1* was barely detectable at mRNA and protein level (Fig. 1A,B). Transcriptome data from a public database confirmed our findings (http://biit.cs.ut.ee/fungenes/). Furthermore, the examination of the abundance of PKD1-3 via immunochemical staining (ICC) indicated predominant cytoplasmic localization of PKD2 in both single ESCs and compact ESC colonies. In line with the mRNA data, PKD1 staining was hardly visible, while PKD3 immunoreactivity confirmed an intermediate expression ranking between PKD1 and PKD2 (Fig. 1C). Accordingly, we next examined the expression of PKD2 during ESC differentiation using embryoid body (EB)-based assays. We observed prominent and significant PKD2 expression peaks at day 2 and day 6 of differentiation on mRNA levels (Fig. 1D). Interestingly, protein levels of PKD2 increased continuously until day 6 (Fig. 1E,G). Of note, the PKD2 antibody used in this study specifically detects PKD2 and does not show cross reactivity with either PKD1 or PKD3^15^. Notably, PKD1 showed moderate protein levels starting at day 4 and day 6 with negligible levels on day 0 and day 2 (Fig. 1E). Next we aimed to assess PKD activity. Western blot analysis with a phospho-specific PKD antibody targeting the activation loop of the kinase demonstrated that undifferentiated ESCs exhibited a moderate basal PKD activity that peaked on day 2 and persisted on lower levels through day 6 of differentiation (Fig. 1F,H). Of note, PKD activity on day 2 mostly reflects PKD2 activity as PKD1 is virtually absent at this stage (Fig. 1B,E). PKD2 specific phosphorylation is further confirmed by immunoprecipitation of the PKD2 protein and subsequent immunoblotting with a phospho-specific PKD antibody (Fig. 1I). PKDs are activated by PKCδ and PKCε, two serine threonine kinases belonging to the novel PKC family^4^,^5^. In mouse embryonic stem cells PKCδ has been shown to be functionally expressed^46^. Indeed, we found phosphorylated PKCδ coincide with PKD2 activity suggesting that in differentiating ESCs, PKCδ could activate PKD2 (Fig. 1J). Together, these data suggest that PKD2 expression and activity may play a role during ESC differentiation. Based on these observations, we decided to focus on PKD2 in further experiments.

### A doxycycline conditional PKD2 knock-in allele

**Figure 2:**
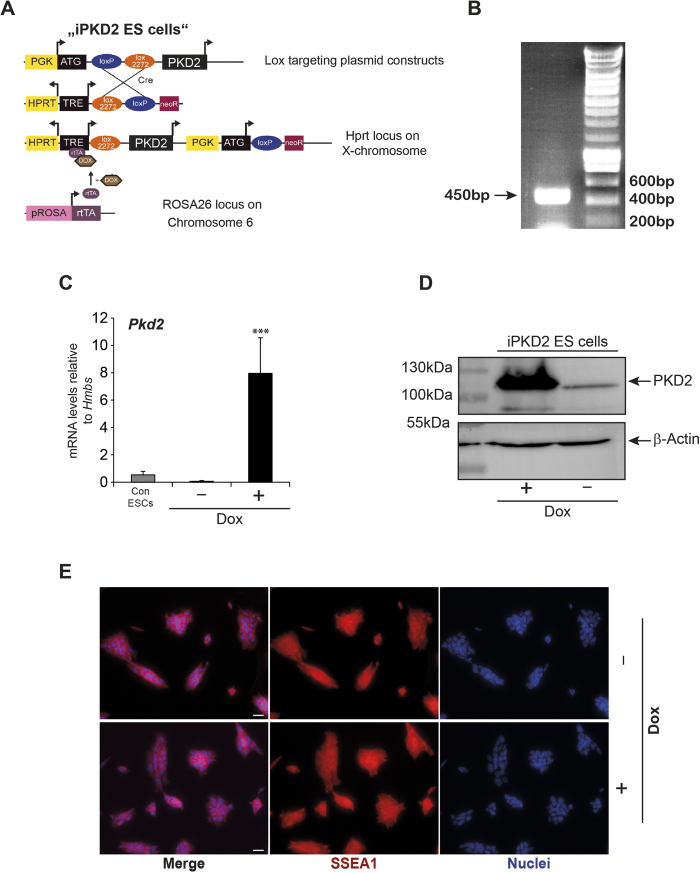
Generation of a conditional PKD2 allele in embryonic stem cells. (**A**) Schematic display of the strategy to generate a dose-dependent Dox-inducible PKD2 ESC line. (**B**) PCR band at 450 bp illustrates the correct recombination of the HPRT locus upon homologous cassette exchange. (**C**) mRNA levels of PKD2 upon Dox-stimulation of iPKD2 ES cells vs. control mES cells (A2lox.cre cell line) (**D**) Western blot of PKD2 upon Dox-stimulation of iPKD2 ES cells. (**E**) Immunostaining of SSEA1 upon Dox induced PKD2 overexpression. Note unaltered expression of SSEA1. qPCRs were performed n = 3 in replicates. Western Blots and immunostaining experiments were performed three times. Scale bars 20 μm.

In order to dissect the role of PKD2 in ESC differentiation, we targeted an inducible PKD2-knock-in allele in ESCs to allow the temporally regulated and dose-dependent expression of PKD2 (iPKD2 ESCs; dose-dependence not shown, Fig. 2A). We used a rapid and efficient recombination system in the HPRT locus, where a doxycycline (Dox)-inducible promoter regulates expression of the PKD2 mRNA. This overexpression system has been extensively applied in a series of studies to delineate the function of a particular gene during differentiation^21^,^39^,^47^,^48^,^49^,^50^,^51^,^52^. Phosphorylation at three critical serine residues of PKD2 has been reported to solely achieve maximum activation (Ser 706, 710 and 244) and not via gene expression alone^4^. Consequently, we targeted a constitutively active PKD2 mutant (PKD2 S244/706/710E, PKD2-3SE) that mimics phosphorylation^4^. The correct gene recombination in the HPRT locus is shown in Fig. 2B. The PKD2 allele was effectively induced upon Dox-exposure as assessed by mRNA and western blot (Fig. 2C,D). Notably, PKD2 overexpression did not affect the expression levels of the SSEA1 protein indicating a negligible role of PKD2 for the pluripotency circuitry (Fig. 2E).

### Timed PKD2 overexpression during ESC differentiation

**Figure 3:**
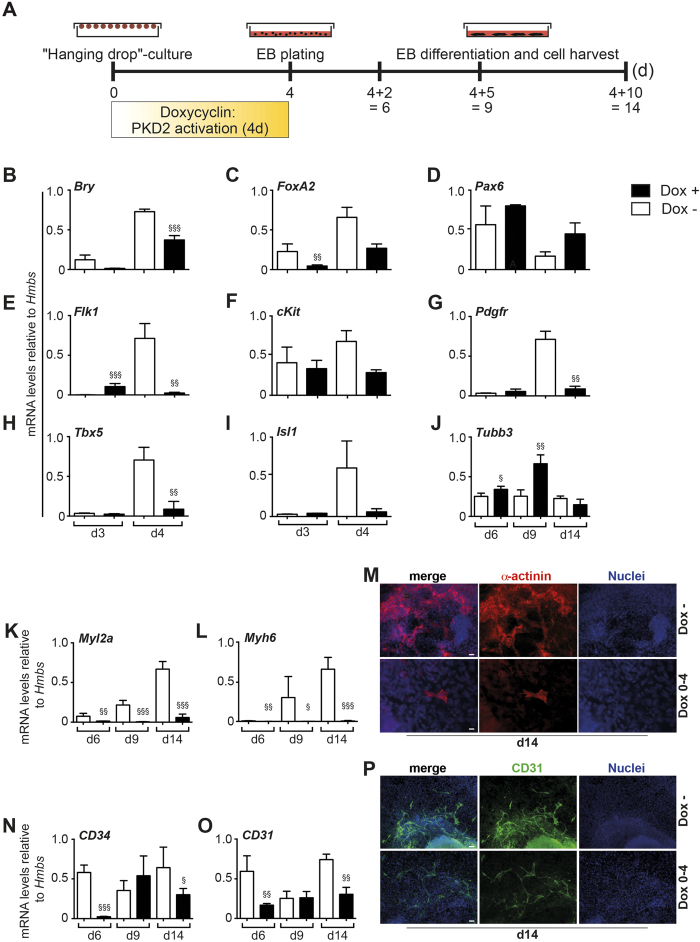
Effects of early PKD2 overexpression from day 0–4. (**A**) Scheme illustrating treatment regimen of iPKD2 ES cells from the beginning of EB culture until cell harvest for qPCR analysis. (**B–D**) qPCR analysis depicting expression levels of different germ layers markers: Mesoderm - Brachyury (Bry); Ectoderm - Pax6; Endoderm -FoxA2. (**E–G**) qPCR analysis illustrating expression of hemangioblast and cardiovascular progenitor markers: hemangioblast/angioblast - Flk1; hemangioblast - c-kit; cardiac -PDGFR. (**H,I,K,L**) qPCR analysis illustrating expression of early cardiac markers, (**H,I**) Tbx5 and Isl1 and late cardiac markers (**K,L**) Myh6, Myl2a. (**J**) Tubb3 marks neuronal differentiation at later time points. (**M**) α-actinin staining of Conditions Dox – and Dox 0–4 at day 14 of differentiation. Cultures were stimulated as illustrated in (**A**). (**N–O**) qPCR analysis illustrating expression of late vascular markers, CD34, CD31 in Dox – and Dox 0–14 conditions. (**P**) CD31 staining at day 14 of differentiation. Cultures were stimulated as illustrated in (**A**). Time points as indicated in the figure and treatment regimen as indicated in the figure. All experiments were performed n = 3 in replicates. Scale bars 20 μm. Significances were calculated using R. Raw p values were adjusted using Bonferroni correction (^§^p < 0.05; ^§§^p < 0.01; ^§§§^p < 0.001). Adjusted p-values are listed in Suppl. Table 1.

In pluripotent stem cells, germ layer segregation occurs around days 2/3^19^,^53^, while the onset of vasculogenesis is ignited around day 3 by the emergence of the hemangioblast, a common progenitor of the endothelial and hematopoietic lineage^54^. The hemangioblast is defined by the co-expression of the vascular endothelial growth factor receptor (Flk1/VEGFR-2) and the mesodermal transcription factor brachyury (T). A second progenitor population arises shortly after the development of the hemangioblast^55^. These cardiovascular progenitors are tripotent giving rise to cardiomyocytes, endothelial cells and smooth muscle cells and co-express Mesp1, brachyury, PDGFRa and c-kit^56^. Using embryoid body (EB) -based differentiation, mimicking early cell fate decision in the embryo, we assessed the effects of overexpression of PKD2 on marker genes of all three germ layers, but also on above mentioned progenitor population markers. In a first approach, PKD2 was activated during the first 4 days of differentiation (Fig. 3A) to investigate changes in germ layer formation and vasculogenesis^54^. In PKD2 overexpressing cells, we observed a downregulation of early markers for mesoderm and endoderm [*Brachyury, FoxA2* (Fig. 3B,C)] and upregulation of the early ectoderm marker *Pax6* (Fig. 3D). In line, markers labelling both the hemangioblast and the tripotent cardiovascular progenitor were repressed [*Flk1*, *cKit*, *Pdgfr* (Figure 3E-G)]. In addition, early cardiac and endothelial markers were downregulated at day 4 [*Tbx5*, *Isl1* (Fig. 3H,I)]. These findings were confirmed with a second, independently targeted iPKD2 ESC line (Supplemental Fig. 1A-F).

Next, we analysed whether late cardiovascular-derived cell types are similarly repressed upon ectopic PKD2 expression in a prolonged ESC differentiation culture. At late time-points of differentiation, EBs overexpressing PKD2 showed diminished cardiac differentiation as demonstrated by the reduced number of beating clusters under the respective culture conditions (data not shown). This was accompanied by decreased mRNA levels of cardiac-specific genes *Myl2a, Myh6* (Fig. 3K,L) and a decreased number of cells expressing α-actinin protein (Fig. 3M). Similarly, endothelial differentiation appeared to be suppressed as shown by downregulated expression of *CD34* and *CD31* mRNA (Fig. 3N,O). CD31 positive vessel-like structures were also reduced in cultures overexpressing PKD2 between day 0 and day 4 (Fig. 3P), the predominant time frame/window when vasculogenesis occurs^54^. β–tubulin 3 (*Tubb3*) expression was enhanced suggesting an inductive effect of PKD2 on the ectodermal germ layer (Fig. 3J). The latter became particularly evident at late time-points of differentiation after PKD2 activation from day 0 to day4 (Fig. 3J, data not shown).

**Figure 4:**
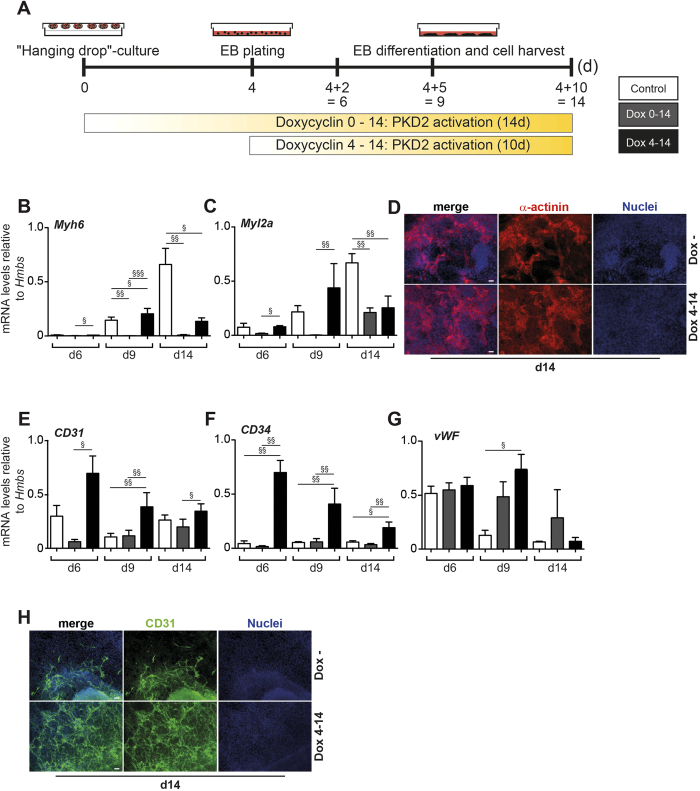
Effects of PKD2 over expression after day 4 of EB development. (**A**) Scheme illustrating treatment regimen of iPKD2 ES cells. (**B**,**C**) mRNA levels of late cardiac markers Myh6 and Myl2a. (**D**) Immunostaining of α-actinin at day 14 of differentiation. (**E–G**) mRNA levels of vascular markers CD31, CD34 and von Willebrand factor (vWF). (**H**) Immunostaining of the vascular protein CD31 in Dox− an Dox 4–14 conditions. Time points and treatment regimen as indicated in the figure. All experiments were performed n = 3 in replicates. Scale bars 20 μm. Significances were calculated using R. Raw p values were adjusted using Bonferroni correction (^§^p < 0.05; ^§§^p < 0.01; ^§§§^p < 0.001). Adjusted p-values are listed in Suppl. Table 2.

The primary vascular plexus in the EB is remodelled by vessel sprouting starting at day 6 and later, thus marking angiogenesis^54^. Given our primary aim to define a PKD2-responsive time-window during vascular development, we triggered the overexpression of PKD2 via Dox-exposure starting at day 4 of differentiation and maintained doxycycline in the culture medium until day 14 of differentiation (day 4 to day 14). Continuous Dox-treatment (day 0 to day 14) was administered to distinguish between an early and late PKD2 responsive window (Fig. 4A). Given the close association between the developmental course of the vascular and cardiac lineage, we next sought to investigate the cardiac differentiation potential. Quantification of beating clusters in differentiating EBs showed no significant difference in cardiac differentiation (data not shown). This observation was further mirrored by the *Myh6*, *Myl2a* gene expression analyses at days 6, 9 and 14 of EB differentiation (Fig. 4B,C) and immunostaining of α-actinin (Fig. 4D). Consistent with an early inhibitory effect of PKD2, when activated from day 0 to day 4, continuous activation led to reduced expression of cardiac markers (Fig. 4B,C). Next, we assessed the development of our primary lineage of interest, namely endothelial cell differentiation. qPCR analysis revealed a significant upregulation of vascular marker genes (*vWF*, *CD34*, *CD31*) in Dox-treated compared to untreated cultures particularly at days 6, 9 and also 14 though less pronounced (Fig. 4E–G). These findings were corroborated with immunostaining for CD31 (Fig. 4H). Of note, *Tubb3* expression was reduced upon later PKD2 induction (data not shown).

### Continuous PKD2 loss-of-function during ESC differentiation

**Figure 5:**
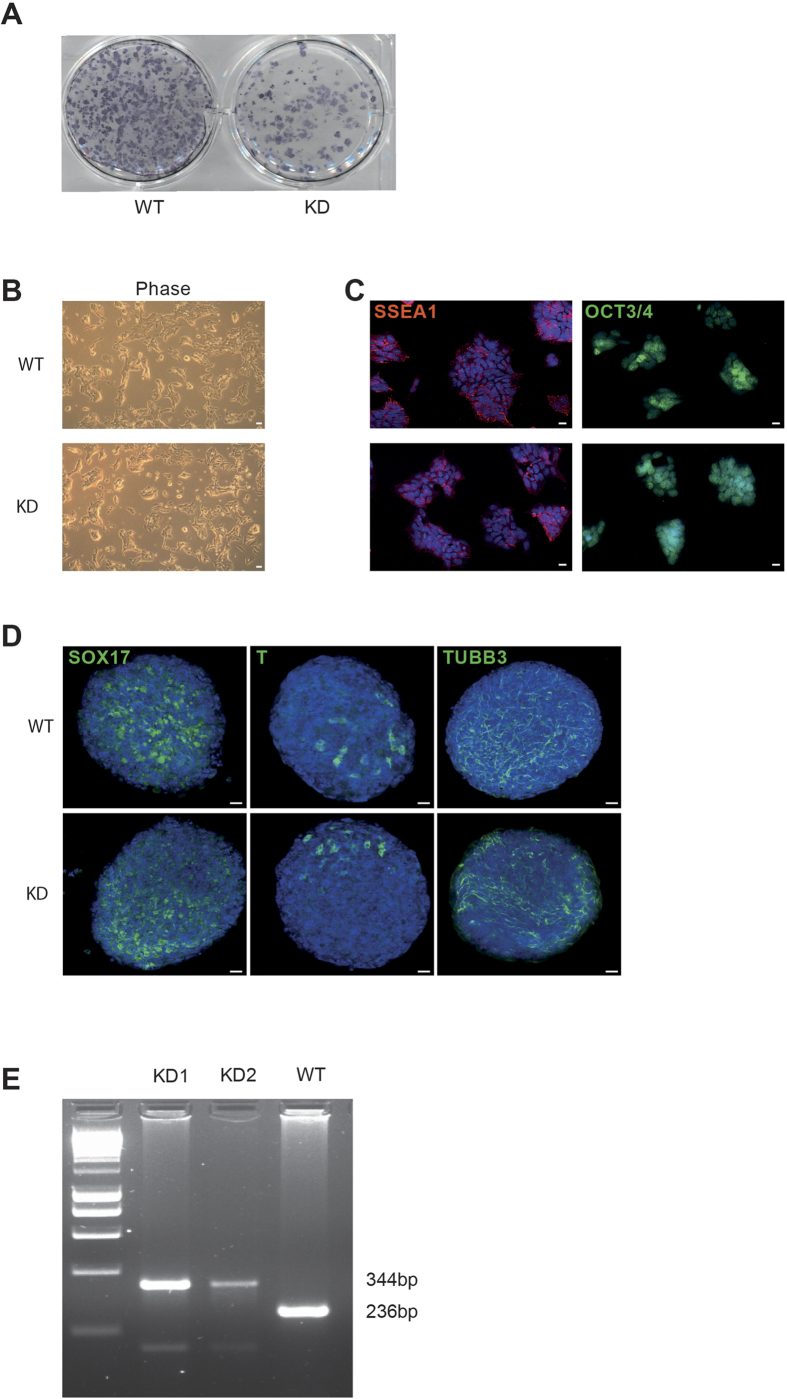
Generation of PKD2 kinase-dead iPS cells. (**A**) AP staining of murine embryonic fibroblasts 13 days after infection with a hOSKM encoding lentivirus. KD = PKD2 kinase-dead; WT = Wildtype. (**B**) Phase contrast microscopy of a feeder-free ESC culture of the respective genotype (**C**) SSEA1 and Oct 3/4 staining of PKD2 kinase-dead (KD)- and wildtype (WT)-iPSCs. (**D**) Whole-mount staining of differentiating embryoid bodies (EBs) under non-pluripotency conditions on day 4. EBS are positive for markers of all three germ layers (Endoderm: Sox17; Mesoderm: T; Ectoderm: Tubb3). (**E**) PCR genotyping of genomic DNA producing products of 236 bp (PKD2-WT) and 344 bp (PKD2 kinase-dead).

**Figure 6:**
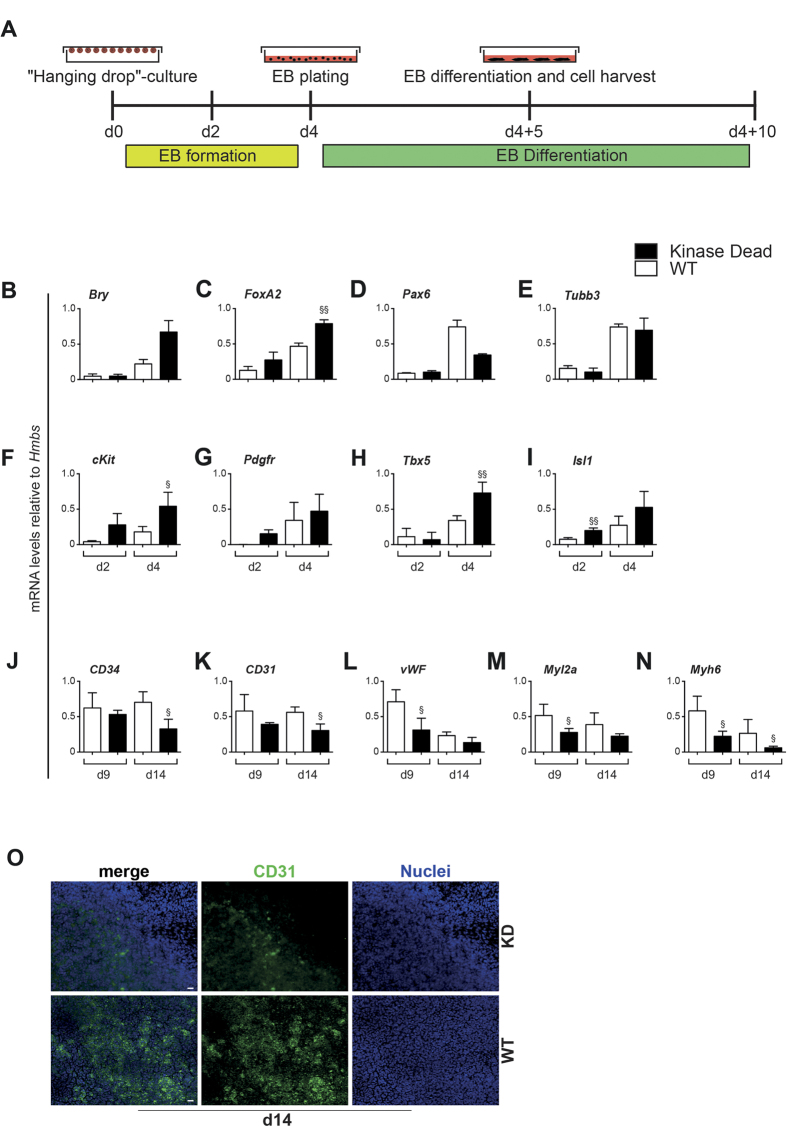
*In vitro* differentiation of PKD2-kinase-dead inactivation. (**A**) Scheme illustrating schedule of EB culture in both PKD2 wild type (WT) and PKD2 kinase-dead (KD) iPSCs. (**B–E**) qPCR analysis depicting expression levels of different germ layers markers: Mesoderm - Brachyury (Bry); Endoderm -FoxA2; Ectoderm -Pax6, -Tubb3. (**F–G**) qPCR analysis illustrating expression of hemangioblast and cardiovascular progenitor markers: c-kit, PDGFR. (**H–I**) qPCR of early cardiac markers: Tbx5 and Isl1. (**J–L**) qPCR analysis of vascular markers CD34, CD31 and vWF. (**M–N**) qPCR of myocardial markers Myl2a and Myh6. (**O**) Immunostaining of differentiating ESC cultures for CD31 at day 14. Genotypes are indicated in the figure. All experiments were performed n = 3 in replicates. Significances were calculated using R. Raw p values were adjusted using Bonferroni correction (^§^p < 0.05; ^§§^p < 0.01; ^§§§^p < 0.001). Adjusted p-values are listed in Suppl. Table 3.

To substantiate the above findings, we made use of a previously reported PKD2 loss-of-function mouse model. In this mouse strain two critical serine residues (i.e. Ser 706, 710) in the kinase domain of PKD2 have been replaced by alanine leading to the expression of a kinase-dead mutant. Therefore, catalytic activity of PKD2 cannot be detected and the mouse recapitulates the phenotype of a PKD2 gene trap line^40^,^41^. Mouse embryonic fibroblasts isolated form this mouse strain served as a somatic template to generate mutant induced pluripotent stem cell lines. Albeit the experiment did not aim to investigate the impact of PKD2 on pluripotency, we noted fewer Alkaline-Phosphatase (AP)-postive cultures in the reprogrammed kinase-dead fibroblasts (Fig. 5A). However, further studies need to focus on this aspect in more detail. We manually selected several induced pluripotent stem cell clones according to their dome-shaped compact colony morphology (Fig. 5B). These iPSC lines displayed all hallmarks of pluripotency such as marker expression of SSEA1 and Oct3/4 (Fig. 5C) and were capable to differentiate into all three germ layers: endoderm (Sox17), mesoderm (T) and ectoderm (Tubb3) (Fig. 5D). The presence of the mutated PKD2 locus was confirmed in two independent clones via PCR (Fig. 5E). Next, we applied two clones to subsequent spontaneous differentiation as outlined in Fig. 6A. qPCR analyses revealed increased mesendodermal marker expression in the early differentiation phase until day 4 (*Brachyury, FoxA2,* Fig. 6B,C), while ectodermal markers *Pax6, Tubb3*) showed decreased expression (Fig. 6D,E). Thus, PKD2-KD iPSCs display the expected, entirely opposite germ layer segregation pattern compared to an early activation of PKD2 (see Fig. 3B–J). Markers of the hemangioblast and the tripotent cardiovascular progenitor showed a similar pattern (Fig. 6F–I). These findings were confirmed with a second PKD2-KD iPSCs clone (Supplemental Fig. 2A-F). Later on, a trend towards decreased terminal differentiation of the cardiovascular lineage was observed as demonstrated by various markers gene expression (Fig. 6J–N). On protein level, CD31 positive cells were fewer and more scattered distributed in PKD2-KD EBs compared to WT, but appeared more organized in structure (data not shown, Fig. 6O).

### PKD2 directs angiogenesis in an *in vivo* model of ESC differentiation

**Figure 7:**
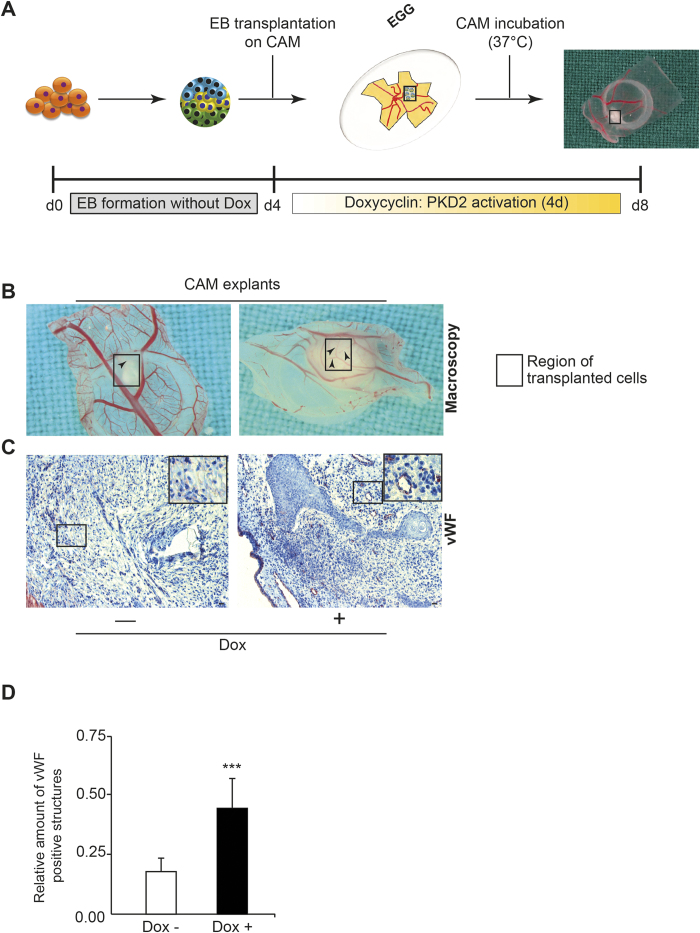
PKD2 driven angiogenesis *in vivo*. Scheme of CAM-assay preparation with EB formation, Dox treatment and EB transplantation. (**B**) Exemplarily display of Dox-treated and untreated CAMs in the unclosed egg. Black square represents region of transplanted EBs. (**C**) Immunohistochemistry for the vWF protein (violet) of Dox-treated and untreated CAMs upon transplantation of EBs. (**D**) Quantification of vWF positive vessels per square under respective conditions on the CAM (for details refer to method section and (**A**)). CAM Assay was performed n = 2. See details in Material and Methods. (**D**) shows the quantification of these experiments.

Finally, we aimed to confirm the basic findings of our data in an *in vivo* model system. The CAM assay is a widely used method to study angiogenesis *in vivo*^57^ and previous studies have already successfully applied this tool to assess EB-derived angiogenesis^58^. Based on our *in vitro* observation that PKD2 induction at later time points of differentiation directs angiogenesis from ESCs, we transplanted untreated 4 days old EBs and subsequently triggered the PKD2 overexpression upon ectopic application of doxycycline onto EBs grafted on the CAM (Fig. 7A). After 5 days, a pronounced sprouting of chicken-derived blood vessels into the tumour-like structures of PKD2-induced EBs was observed (Fig. 7B). Moreover, we observed more vessel-like structures inside the differentiating EBs compared to untreated EBs, as demonstrated by enhanced immunoreactivity for the vWF protein (Fig. 7C,D).

## Discussion

Previous studies have shown distinct but also overlapping functions of the different PKD family members (PKD1-3) during mouse embryogenesis^11^,^12^. PKD1 kinase-dead knock-in mice die *in utero* around day E9.5 while mice featuring a PKD2 deletion are viable and fertile^40^,^41^. However, a detailed phenotypic analysis of this early lethality in PKD1 null remains elusive but primitive haematopoiesis and vasculogenesis start around this time period. PKD2 exhibits unique characteristics among the PKD family members, due to its pronounced role during physiological and tumour angiogenesis. Yet, implications of PKD2 during cell fate determination of embryonic stem cells remain elusive. Moreover, despite an intimate connection between vasculogenesis and angiogenesis during early development, currently available knowledge is limited to the role of PKD2 during angiogenesis. The current study addresses this by the application of mouse embryonic stem cell differentiation using both complementary gain- and loss-of-function approaches. Thereby we are able to define a time-responsive window being accessible for PKD2 activity and show that PKD2 limits mesendodermal commitment and vasculogenesis but later on induces branching angiogenesis.

Interestingly, the three PKD isoforms act in a context- and cell-type dependent, similar or opposing manner^17^,^31^: on the one hand, PKD2 gain-of-function in pancreatic cancer promotes invasion and angiogenesis while PKD1 limits these processes^31^; on the other hand, angiogenesis in the CAM is similarly promoted by the PKD isoforms^29^. Moreover, PKD1 and 2 share various structural and functional similarities while PKD3 is not as homologous to the two other family members. Particularly, the N-terminus of both PKD1 and PKD2 starts with a hydrophobic domain, which is absent in PKD3. Also, PKD3 lacks the autophosphorylation sites at S916/S876, which have been shown to regulate PKD conformation and activation length (for review see^2^). Herein, our own data obtained in skeletal muscle stem cells, so-called myoblasts, indicate PKD2 to be expressed at considerably higher levels than PKD1. In line, PKD2-depleted myoblasts exhibited impaired, while forced-PKD2 expression increased myoblast differentiation. Of note, PKD1 and PKD3 had only minor effects. This observation indicates that assessing expression levels of PKDs in the parental stem cell population can predict its relevance during differentiation^15^. This together with the fact that PKD1 was virtually undetectable in undifferentiated ESCs, prompted us to focus on PKD2 in the current study. Intriguingly, atypical PKCs direct differentiation of human and mouse embryonic stem cells leading to PKC inhibitor based culture systems to keep pluripotent stem cells in an undifferentiated stage^45^,^59^. While we did not observe an obvious impact upon forced PKD2 expression on pluripotency, we noticed fewer AP positive areas in EBs derived from iPSCs expressing the kinase-dead mutant. This urges for further detailed investigations of PKD2 as one possible subordinate player to affect pluripotency.

*De novo* blood vessel formation defines vasculogenesis while formation of sprouting blood vessels from pre-existing vessels is commonly considered as angiogenesis. While embryonic vasculogenesis is difficult to model *in vitro*, common adult angiogenesis models to study e.g. formation of vessel tube formation comprise primary endothelial cells or endothelial cell lines such as HUVECs. Interestingly, both embryonic processes can be studied in differentiating embryonic stem cells^54^,^60^, a unique feature of ESCs not offered by any other cellular model system. Differentiating ESCs are not only able to display vasculogenic features during the first 4 days of EB differentiation. At later stages, after formation of early blood vessels/-angioblasts, they also exhibit embryonic branching angiogenesis. Thus, a particular factor can be investigated with respect to its impact on both processes in the same culture system^54^,^60^. Nevertheless, pure *in vitro* vasculogenesis and angiogenesis models lack complex processes that e.g. require progression through several developmental stages^54^. For that, we made use of a combined *in vitro/in vivo* CAM (chorioallantoic membrane) assay. This assay comprises the advantages of physiological vessel formation with an easy and fast possibility to transplant embryoid bodies derived from gene-manipulated, differentiating ESCs.

To integrate these advantages into one system, we engineered an inducible knock-in allele targeting PKD2 to the HPRT locus and complemented this tool with a genetic loss-of-function system, the PKD2 kinase-dead expressing iPSCs^21^,^39^,^47^,^49^,^50^. To our surprise, early PKD2 activation did not induce vasculogenesis, but rather repressed mesendodermal differentiation, while inducing a neuroectodermal fate. In line, abrogation of PKD2 activity using genetic loss-of-function tools in pluripotent stem cells triggered opposing effects mirrored by the induction of mesendodermal derivatives and cardiovascular progenitors in expense of a neuroectodermal fate. This is particularly interesting in light of our data showing a PKD2 activation peak around day 2 of differentiation. At this stage, PKD1 remains virtually undetectable. On the other hand, we observed an induction of vascular networks in late PKD2 overexpressing cultures, when PKD2 was induced after day 4 of differentiation. This period coincides with embryonic branching angiogenesis in the ESC-based EB differentiation system^54^,^60^. To our surprise, there was a trend towards reduced terminal cardiovascular differentiation albeit only some markers turned out to be significantly changed, despite the fact that the mesendodermal germ layer was induced. This discordance between gain-of-function and loss-of-function data might be explained by the inability of timed manipulation in the PKD2 loss-of-function system. Spatiotemporal differences in PKD2 function/requirement during embryonic stem cell differentiation, as suggested by our gain-of-function data, could attenuate the inductive effect of PKD2 loss after germ layer segregation has occurred. However, the CD31 positive vascular structures appeared, despite overall reduced, more branched compared to control cultures. This observation needs further investigation in future studies.

How could these phenotypic observations be explained? Several reports suggest PKD2 to act as the predominant isoform promoting physiologic and tumour angiogenesis^26^. Second, the PKD2 knock-out mouse is viable and fertile without any gross abnormalities, thus the catalytic activity appears not to be crucial for *in vivo* mouse embryogenesis^41^, (see below). In line, knock-out mouse models of genes being highly critical for proper vasculogenesis, usually undergo embryonic lethality^61^,^62^,^63^. Thus, our data showing enhanced mesendodermal commitment upon PKD2 loss is consistent with a viable PKD2 knock-out mouse. Third, a recent manuscript using the zebrafish as model system reported only minimal changes with respect to vasculogenesis, but showed strong effects when PKD1 was knocked-out during xenograft angiogenesis^33^. Fourth, a loss-of-function approach in Drosophila showed wing vein defects, but no other vascular malformations were reported^64^.

However, there is still a remaining discordance between a viable and fertile PKD2 knock-out mouse and our *in vitro* data reporting alterations during germ layer formation and cardiovascular differentiation. We cannot exclude that compensational events might mask various effects during development in case of loss of a particular PKD isoforms. Indeed, overlapping expression between PKD1, 2 and 3 have been reported in several studies. In particular, PKD1 and PKD2 isoforms display very similar structural properties and phenotypes. Briefly, our own group has gathered deep insights into PKD isoform specificity and defined various overlapping functions in different cancers types including control of proliferation, angiogenesis or cell motility^2^,^29^,^30^,^65^,^66^,^67^,^68^ and differential expression in various stem cell populations^15^. Of note, we recently reported PKD actions with isoform specific behaviour and even opposing effects^15^,^31^. In fact, specific events such as gastrulation but also vasculogenesis leading to early lethality in the developing embryo are more frequently protected. Thus, compensation is an established issue throughout development and differentiation^69^,^70^,^71^,^72^,^73^,^74^,^75^,^76^,^77^, particularly of vascular development^78^,^79^,^80^,^81^. Apparently this compensation is attenuated in our *in vitro* system, improving the chances to identify factor specific functions^21^ and isoform specific effects of a particular protein family^21^.

In summary, the current study suggests a role of PKDs during embryonic development using a complementary gain and loss-of-function approach. For the first time, we can show that PKD2 limits mesendoderm formation and cardiovascular progenitor formation, but preformed vessels arising after vasculogenesis become permissive for PKD2 activity to allow embryonic branching angiogenesis. Still, there are remaining questions to be answered to gather deeper insights into the complex isoform specific interplay of PKDs during development. Conditional but also combinational knock-out mouse models complemented with tightly to regulate gain- and loss-of-function *in vitro* systems will support future studies.

## Additional Information

**How to cite this article**: Müller, M. *et al.* A time frame permissive for Protein Kinase D2 activity to direct angiogenesis in mouse embryonic stem cells. *Sci. Rep.* **5**, 11742; doi: 10.1038/srep11742 (2015).

## Supplementary Material

Supplementary Information
