# Supplementary material for: A time frame permissive for Protein Kinase D2 activity to direct angiogenesis in mouse embryonic stem cells
Source: Sci Rep. 2015 Jul 7;5:11742. doi: 10.1038/srep11742 (PMC4493579; doi:10.1038/srep11742)
Supplement: Supplementary Information [file srep11742-s1.pdf]

A time frame permissive for Protein Kinase D2 activity to direct angiogenesis in mouse embryonic stem cells

Martin Müller[1*#], Jana Schröer[1*], Ninel Azoitei[1], Tim Eiseler[1], Wendy Bergmann[1], Ralf Köhntop[1], Qiong Lin[2], Ivan G Costa[4], Martin Zenke[2], Felicitas Genze[3], Clair Weidgang[1], Thomas Seufferlein[1], Stefan Liebau[5#], Alexander Kleger[1#]

[1] Department of Internal Medicine I, Ulm University, Ulm, Germany

[2] Department of Cell Biology, Institute for Biomedical Engineering, Medical Faculty, RWTH University Aachen, Germany

[3] Department of Urology, Ulm University, Ulm, Germany

[4] IZKF Computational Biology Research Group, RWTH Aachen University Medical School, Germany

[5] Institute of Neuroanatomy, Eberhard Karls University Tuebingen, Tuebingen, Germany

Supplemental Figure Legends

Supplemental Figure 1: Effects of early overexpression in a second independently targeted iPKD2 ESC line. The generation of this second iPKD2 cell line was performed according to **Figure 2A**. Cultures were stimulated as illustrated in **Figure 3A**. Expression levels of (**A**) FoxA2, (**B**) Pax6, (**C**) Flk1, (**D**) cKit, (**E**) Isl1 and (**F**) Tbx5 on day 4 of embryoid body differentiation are shown. Supplemental Experiments were performed n=2.

Supplemental Figure 2: Differentiation of PKD2-kinase dead EBs derived from a second iPSC clone. These data were generated from two clones (WT and KD) further expanded after picking from reprogramming MEF cultures from the same genotype. Regulation of (**A**) FoxA2, (**B**) Pax6, (**C**) Pdgfr, (**D**) cKit, (**E**) Isl1 and (**F**) Tbx5 on day 4 of differentiation is shown. EB differentiation was performed according to **Figure 6A.** KD: kinase dead, WT: wild type. Supplemental Experiments were performed n=2.

Supplemental Table 1

List of adjusted p-values for Figure 3

Supplemental Table 2

List of adjusted p-values for Figure 4

Supplemental Table 3

List of adjusted p-values for Figure 6

Supplemental Figure 1

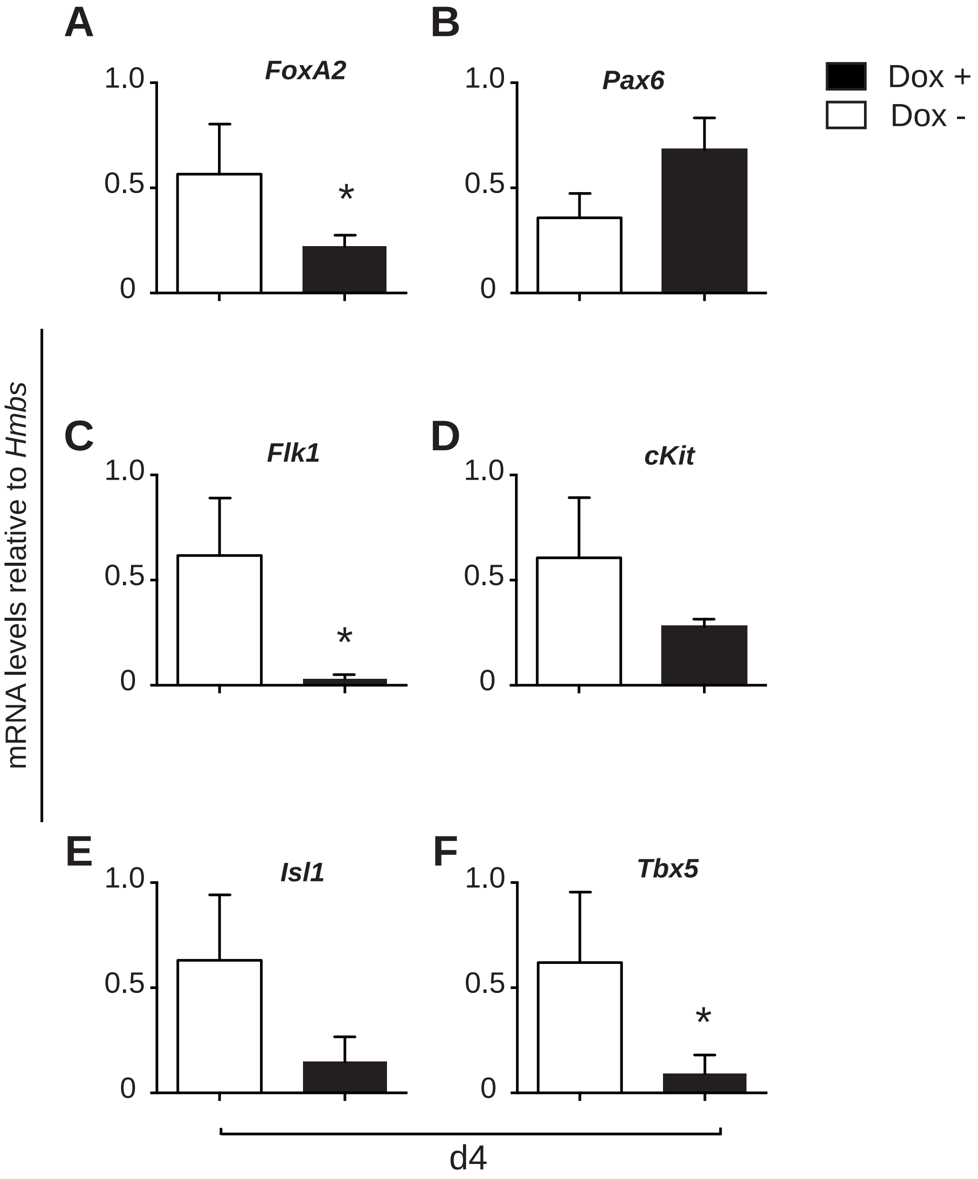

Supplemental Figure 2

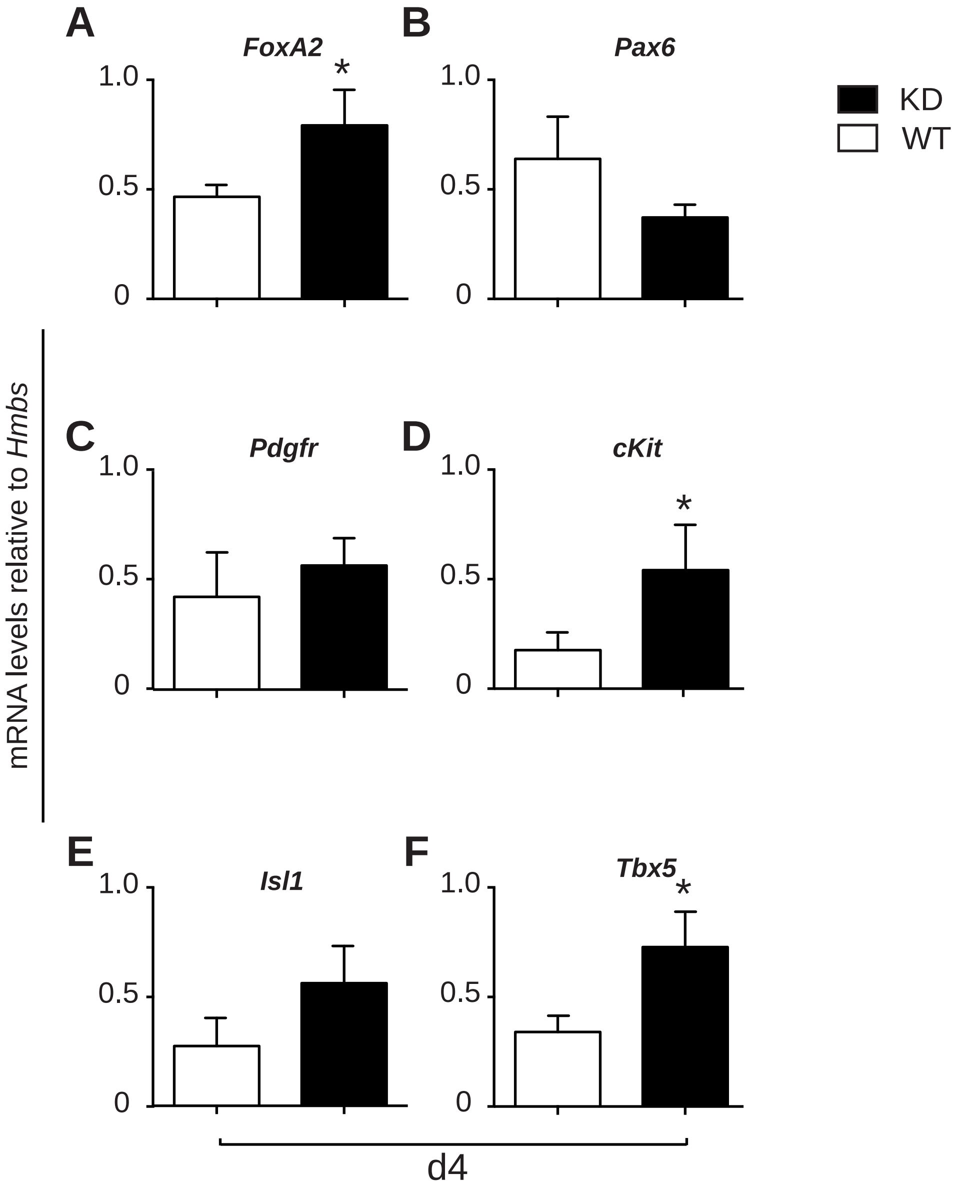

Time point	Gene	p-value (two-side t-test)	adjusted p-value (Bonferroni multiple correction)	Figure
D3	Bry	0,059481475	0,083221214	3B
D4	Bry	**7,70395E-05**	**0,000693356**	3B
D3	FoxA2	**0,00020887**	**0,001009031**	3C
D4	FoxA2	**0,023789172**	0,051177564	3C
D3	Pax6	0,801834803	0,849001556	3D
D4	Pax6	**0,027426034**	0,051177564	3D
D3	Flk1	**6,97731E-06**	**0,000125592**	3E
D4	Flk1	**0,002290388**	**0,005889569**	3E
D3	cKit	0,587340367	0,660757913	3F
D4	cKit	**0,032948463**	0,053915667	3F
D3	PDGFR	0,06010421	0,083221214	3G
D4	PDGFR	**0,000425008**	**0,001381395**	3G
D3	Tbx5	0,896096854	0,896096854	3H
D4	Tbx5	**0,000460465**	**0,001381395**	3H
D3	Isl1	0,193981782	0,249405148	3I
D4	Isl1	**0,02843198**	0,051177564	3I

Time point	Gene	p-value (two-side t-test)	adjusted p-value (Bonferroni multiple correction)	Figure
D6	Tubb3	**0,02071165**	**0,02867767**	3J
D9	Tubb3	**0,000976387**	**0,00210614**	3J
D14	Tubb3	0,147065741	0,16544900	3J
D6	Myl2a	**0,000437502**	**0,00157501**	3K
D9	Myl2a	**4,01256E-06**	**0,00005283**	3K
D14	Myl2a	**2,53933E-05**	**0,00015236**	3K
D6	Myh6	**0,00072598**	**0,00210614**	3L
D9	Myh6	**0,018740047**	**0,02811007**	3L
D14	Myh6	**8,08518E-05**	**0,00036383**	3L
D6	CD34	**5,86989E-06**	**0,00005283**	3N
D9	CD34	0,112694398	0,14489280	3N
D14	CD34	**0,011219655**	**0,01835944**	3N
D6	CD31	**0,001644655**	**0,00296038**	3O
D9	CD31	0,258172333	0,27335890	3O
D14	CD31	**0,001053071**	**0,00210614**	3O

Time point	Compared Pair	Gene	p-value (Anova PostHoc)	adjusted p-value (Bonferroni multiple correction)	Figure
D6	Control vs. Dox 0-14	Myh6	0,1322842	0,210098435	4B
D6	Control vs. Dox 4-14	Myh6	0,5332273	0,625962483	4B
D6	Dox 4-14 vs. Dox 0-14	Myh6	**0,0170378**	**0,040001791**	4B
D9	Control vs. Dox 0-14	Myh6	**0,000227**	**0,0030645**	4B
D9	Control vs. Dox 4-14	Myh6	**0,0212022**	**0,04770495**	4B
D9	Dox 4-14 vs. Dox 0-14	Myh6	**0,0000014**	**0,0000756**	4B
D14	Control vs. Dox 0-14	Myh6	**0,000299**	**0,0032292**	4B
D14	Control vs. Dox 4-14	Myh6	**0,00765**	**0,018777273**	4B
D14	Dox 4-14 vs. Dox 0-14	Myh6	0,2574168	0,356423262	4B
D6	Control vs. Dox 0-14	Myl2a	0,0517894	0,096435434	4C
D6	Control vs. Dox 4-14	Myl2a	0,5085178	0,61933524	4C
D6	Dox 4-14 vs. Dox 0-14	Myl2a	**0,0055507**	**0,015775674**	4C
D9	Control vs. Dox 0-14	Myl2a	**0,0246724**	0,053292384	4C
D9	Control vs. Dox 4-14	Myl2a	**0,0351163**	0,067724293	4C
D9	Dox 4-14 vs. Dox 0-14	Myl2a	**0,0001081**	**0,0025758**	4C
D14	Control vs. Dox 0-14	Myl2a	**0,0001431**	**0,0025758**	4C
D14	Control vs. Dox 4-14	Myl2a	**0,00132**	**0,00594**	4C
D14	Dox 4-14 vs. Dox 0-14	Myl2a	0,4939166	0,61933524	4C
D6	Control vs. Dox 0-14	CD31	0,5161127	0,61933524	4E
D6	Control vs. Dox 4-14	CD31	**0,0299535**	0,0621442	4E
D6	Dox 4-14 vs. Dox 0-14	CD31	**0,0032149**	**0,010212035**	4E
D9	Control vs. Dox 0-14	CD31	0,9861775	0,9996584	4E
D9	Control vs. Dox 4-14	CD31	**0,0004214**	**0,003519257**	4E
D9	Dox 4-14 vs. Dox 0-14	CD31	**0,0005724**	**0,0038637**	4E
D14	Control vs. Dox 0-14	CD31	0,2320964	0,3298212	4E
D14	Control vs. Dox 4-14	CD31	0,1699757	0,262248223	4E
D14	Dox 4-14 vs. Dox 0-14	CD31	**0,006621**	**0,017336829**	4E
D6	Control vs. Dox 0-14	CD34	0,9691476	0,9996584	4F
D6	Control vs. Dox 4-14	CD34	**0,0015762**	**0,006547292**	4F
D6	Dox 4-14 vs. Dox 0-14	CD34	**0,0009848**	**0,004853127**	4F
D9	Control vs. Dox 0-14	CD34	0,9996584	0,9996584	4F
D9	Control vs. Dox 4-14	CD34	**0,0009416**	**0,004853127**	4F
D9	Dox 4-14 vs. Dox 0-14	CD34	**0,0009886**	**0,004853127**	4F
D14	Control vs. Dox 0-14	CD34	0,6065573	0,696895621	4F
D14	Control vs. Dox 4-14	CD34	**0,0030665**	**0,010212035**	4F
D14	Dox 4-14 vs. Dox 0-14	CD34	**0,0004562**	**0,003519257**	4F
D6	Control vs. Dox 0-14	vWF	0,6416134	0,721815075	4G
D6	Control vs. Dox 4-14	vWF	0,3461266	0,46727091	4G
D6	Dox 4-14 vs. Dox 0-14	vWF	0,8582981	0,926961948	4G
D9	Control vs. Dox 0-14	vWF	0,2003191	0,297414924	4G
D9	Control vs. Dox 4-14	vWF	**0,0037149**	**0,0111447**	4G
D9	Dox 4-14 vs. Dox 0-14	vWF	0,122413	0,200312182	4G
D14	Control vs. Dox 0-14	vWF	0,0806137	0,140423865	4G
D14	Control vs. Dox 4-14	vWF	0,9757157	0,9996584	4G
D14	Dox 4-14 vs. Dox 0-14	vWF	0,1174439	0,198186581	4G

Time point	Gene	p-value (two-side t-test)	adjusted p-value (Bonferroni multiple correction)	Figure
D2	Bry	0,926812518	0,926812518	6B
D4	Bry	0,926812518	0,926812518	6B
D2	FoxA2	0,083579763	0,148586245	6C
D4	FoxA2	**0,001387437**	**0,008184587**	6C
D2	Pax6	0,801834803	0,916382632	6D
D4	Pax6	**0,027426034**	0,087763309	6D
D2	Tubb3	0,060893021	0,121786042	6E
D4	Tubb3	0,318056623	0,427560057	6E
D2	cKit	**0,048032543**	0,10978867	6F
D4	cKit	**0,004197417**	**0,016789668**	6F
D2	PDGFR	**0,042149463**	0,10978867	6G
D4	PDGFR	0,335996041	0,427560057	6G
D2	Tbx5	0,347392546	0,427560057	6H
D4	Tbx5	**0,00153461**	**0,008184587**	6H
D2	Isl1	**0,001330794**	**0,008184587**	6I
D4	Isl1	0,118332618	0,189332189	6I

Time point	Gene	p-value (two-side t-test)	adjusted p-value (Bonferroni multiple correction)	Figure
D9	CD34	0,382383352	0,38238335	6J
D14	CD34	**0,009067221**	**0,02731819**	6J
D9	CD31	0,056244612	0,07030576	6K
D14	CD31	**0,012394669**	**0,02731819**	6K
D9	vWF	**0,001142494**	**0,01142494**	6L
D14	vWF	0,055140116	0,07030576	6L
D9	Myl2A	**0,005073762**	**0,02536881**	6M
D14	Myl2A	0,065125459	0,07236162	6M
D9	MyH6	**0,01632989**	**0,02731819**	6N
D14	MyH6	**0,016390913**	**0,02731819**	6N
